# Folic Acid Supplementation Suppresses Sleep Deprivation-Induced Telomere Dysfunction and Senescence-Associated Secretory Phenotype (SASP)

**DOI:** 10.1155/2019/4569614

**Published:** 2019-12-14

**Authors:** Xiaoning Zhang, Yuwen Wang, Rui Zhao, Xianyun Hu, Baoren Zhang, Xin Lv, Zhenglong Guo, Zhiqiang Zhang, Jinghua Yuan, Xu Chu, Fei Wang, Guang Li, Xin Geng, Yang Liu, Lei Sui, Feng Wang

**Affiliations:** ^1^Department of Genetics, School of Basic Medical Sciences, Tianjin Medical University, Tianjin 300070, China; ^2^Institute of Basic Medicine, Shandong First Medical University & Shandong Academy of Medical Sciences, Shandong 250012, China; ^3^Department of Medical Examination, Tianjin Worker's Hospital, Tianjin 300050, China; ^4^Department of General Surgery, Tianjin General Surgery Institute, Tianjin Medical University General Hospital, Tianjin 300052, China; ^5^Department of Nutrition and Food Science, School of Public Health, Tianjin Medical University, Tianjin 300070, China; ^6^Department of Cell Biology, School of Basic Medical University, Tianjin Medical University, Tianjin 300070, China; ^7^Department of Pathology, Tianjin Hospital of ITCWM, Nankai Hospital, Tianjin 300100, China; ^8^School of Medicine, Hangzhou Normal University, Zhejiang 310036, China; ^9^Department of Neurology, General Hospital, Tianjin Medical University, Tianjin 300052, China; ^10^Department of Biochemistry and Molecular Biology, School of Basic Medical Sciences, Tianjin Medical University, Tianjin 300070, China; ^11^Department of Radiobiology, Institute of Radiation Medicine, Chinese Academy of Medical Sciences and Peking Union Medical College, Tianjin 300192, China; ^12^Department of Prosthodontics, Tianjin Medical University School and Hospital of Stomatology, Tianjin 300070, China; ^13^Tianjin Key Laboratory of Exercise Physiology and Sports Medicine, Tianjin University of Sport, Tianjin 300381, China

## Abstract

Sleep deprivation is reported to cause oxidative stress and is hypothesized to induce subsequent aging-related diseases including chronic inflammation, Alzheimer's disease, and cardiovascular disease. However, how sleep deprivation contributes to the pathogenesis of sleep deficiency disorder remains incompletely defined. Accordingly, more effective treatment methods for sleep deficiency disorder are needed. Thus, to better understand the detailed mechanism of sleep deficiency disorder, a sleep deprivation mouse model was established by the multiple platform method in our study. The accumulation of free radicals and senescence-associated secretory phenotype (SASP) was observed in the sleep-deprived mice. Moreover, our mouse and human population-based study both demonstrated that telomere shortening and the formation of telomere-specific DNA damage are dramatically increased in individuals suffering from sleeplessness. To our surprise, the secretion of senescence-associated cytokines and telomere damage are greatly improved by folic acid supplementation in mice. Individuals with high serum baseline folic acid levels have increased resistance to telomere shortening, which is induced by insomnia. Thus, we conclude that folic acid supplementation could be used to effectively counteract sleep deprivation-induced telomere dysfunction and the associated aging phenotype, which may potentially improve the prognosis of sleeplessness disorder patients.

## 1. Introduction

Sleep is a physiological state that is vital for the quality of life of an individual and occupies 1/4-1/3 of the time in one day in most humans. During sleep, most of the body's systems are in a state of synthesis, helping to restore the immune, nervous, skeletal, and muscular systems, which are important to maintain emotion, memory, and cognitive functions [1]. However, sleep deprivation (SD) or chronic sleep restriction has become a relevant health problem caused by social factors, such as wide usage of electronic products and networks, night-shift work or overtime work schedules, and chronic diseases [2–4]. Previous studies have shown that sleep deprivation leads to a number of aging-related diseases, including chronic inflammation, Alzheimer's disease, and cardiovascular disease [5–10] and even causes mortality when individuals are severely deprived of sleep [11, 12].

A main cause of sleep deprivation-induced disease is increased oxidative damage [13]. Oxidative stress is a phenomenon caused by an imbalance between the production and accumulation of reactive oxygen species (ROS) in cells and tissues and the ability of a biological system to detoxify these reactive products [14]. A large number of studies have shown that oxidative stress and increased ROS production could lead to cell trauma and cause several diseases [14–18].

Telomeres are a type of repetitive nucleotide sequence (5′-(TTAGGG)_*n*_-3′) at the ends of all chromosomes that protect the chromosome from deterioration or fusion with neighboring chromosomes. Due to their high guanine content, telomeres are considered common fragile sites of the genome and are highly sensitive to damage and are shortened by chronic oxidative stress, toxin damage, and radiation exposure [19, 20]. When telomeres become critically short or sufficiently damaged, DNA damage signaling is triggered, leading to various senescence-related disorders [10].

Folate, also known as the natural form of vitamin B9 in food, which is the generic term for a family of compounds including folic acid (a synthetic, parent compound of this family which does not exist in nature) and its derivatives is a critical cofactor in one-carbon metabolism, including nucleotide metabolism, maintaining the cellular redox status and methylation metabolism [21, 22]. Folate is also required for nucleotide synthesis and is implicated in cell proliferation, DNA repair, and genomic stability [23]. Current studies have shown that folic acid functions in the treatment of neural tube defect (NTD), cardiovascular disease (CVD), and stroke [24–26].

In this study, we used a multiple classical platform technique to investigate the hypothesis that folic acid supplementation prevents sleep deprivation-induced oxidative stress and telomere dysfunction in an animal model. Furthermore, we recruited human volunteers to investigate the correlation between sleep quality, folic acid, and telomere length.

## 2. Materials and Methods

### 2.1. Animal Experiments

#### 2.1.1. Animals and Sleep Deprivation

Wild-type male C57BL/6J mice (4~5 weeks) were purchased from Huafukang Bioscience Co., Inc. (BJ, CN). Mice were randomly divided into two groups with similar mean body weights (14 mice per group): the FAD group (mice were fed a folic acid-deficient diet) and the FAS group (mice were fed a folic acid-supplemented diet).

**Figure 1:**
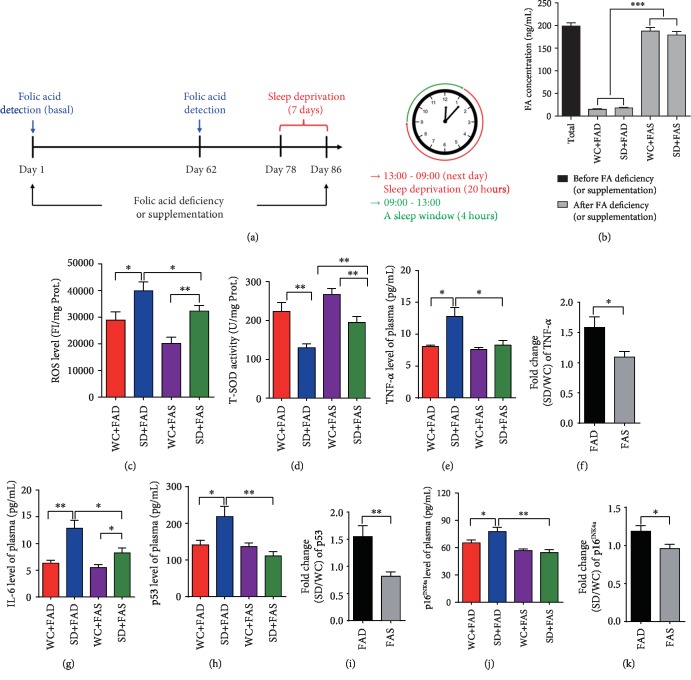
Folic acid attenuates sleep deprivation-induced oxidative stress and SASP disorder. (a) Experimental design. A schematic diagram showing that mice were fed a long-term folic acid-supplemented diet for more than two months, and sleep deprivation was performed for 20 h/day (01:00 pm to 09:00 am the following day) for 7 days. (b) The folic acid concentration in serum before and after folic acid deficiency (or supplementation). (c) Reactive oxygen species (ROS) in the liver was assayed using DCFH-DA as a probe. (d) Antioxidative capacity in the liver was detected by the total SOD assay kit with WST-8. (e, f) Secretion of tumor necrosis factor-*α* (TNF-*α*) in the plasma, detected by ELISA, and the fold change (SD/WC) in the FAD or FAS groups. (g) Secretion of interleukin-6 (IL-6) in the plasma, detected by ELISA. (h–k) The abundance of p53 and p16^INK4a^ in the plasma was detected by ELISA and the fold change (SD/WC) in the FAD or FAS groups. Data are presented as the mean ± SEM (*N* = 7 per group). ^∗^*P* < 0.05; ^∗∗^*P* < 0.01; and ^∗∗∗^*P* < 0.001.

All mice were fed one purified diet based on the AIN93M standard formula [27] without the addition of folic acid (FAD) or with the addition of 8 mg/kg folic acid (FAS) (for the composition of the experimental diets, see Supplementary [Supplementary-material supplementary-material-1]). All diets were manufactured by Trophic Animal Feed High-Tech Co., Ltd. (JS, CN). Before sleep deprivation, mice were fed a folic acid-deficient or folic acid-supplemented diet for 78 days. FAD and FAS mice were both randomly subdivided into two groups: wide platform control groups (WC+FAD and WC+FAS) and sleep deprivation groups (SD+FAD and SD+FAS) (seven mice per group). Mice were sleep deprived using the multiple classical platform technique, as described previously (the experimental design is shown in Figure 1(a)) [28, 29]. Briefly, mice in a group were placed on narrow platforms (*Φ* 3 cm, 12 platforms per container) in a water tank (41 cm × 34 cm × 17 cm) surrounded by water up to 1 cm beneath the surface (schematic photo in Supplementary Figures [Supplementary-material supplementary-material-1] and [Supplementary-material supplementary-material-1]). In this method, mouse sleep was disrupted when the mice fell into the water after muscle atonia. The duration of sleep deprivation was 20 h, which ran from 1:00 pm to 9:00 am the following day, and was conducted over 7 days; the mice slept for 4 h every day. Control group mice were placed on a large platform (*Φ* 6 cm, 8 platforms per container) in the water tank where the mice were able to sleep and move at will. Mice were maintained under specific pathogen-free conditions (temperature controlled 22.5 ± 0.5°C) with a 12-12 h light-dark cycle (lights were turned on at 7:30 am and turned off at 7:30 pm) according to institutional guidelines. Experimental protocols and animal care methods were approved by the Animal Care and Use Committee of Tianjin Medical University (TMUaMEC 2017012).

#### 2.1.2. Folate Concentration Assays

Serum folate concentration was determined by a competitive protein-binding assay with a Siemens automated chemiluminescence system (BER, DE), according to the manufacturer's instructions. The detectable concentration ranged from 1 to 24 ng/mL, since the serum folate levels were high, and the samples were diluted by a factor of 5 to 20 with normal saline.

#### 2.1.3. ROS Levels and SOD Activity Assays

The liver and testis were homogenized (1 : 20, *w*/*v*) in ice-cold phosphate-buffered saline (PBS). The homogenate was centrifuged at 9000 ×g at 4°C for 15 min to remove cell debris. The supernatant was harvested and incubated with dichlorodihydrofluorescein diacetate (DCFH-DA) (Wanlei Life Sciences Co., Ltd., LN, CN) at 37°C for 30 min to assess intracellular ROS generation. Fluorescence was examined using a Multifunctional Enzyme Marker (Tecan, Männedorf, CH) at excitation/emission wavelengths of 485/525 nm. Total SOD activity was detected by a Total SOD Assay Kit with WST-8 (Wanlei Life Sciences Co., Ltd.) according to the manufacturer's instructions. The absorbance was assessed at 550 nm using a Microplate Reader (Bio-Rad, CA, USA).

#### 2.1.4. Enzyme-Linked Immunosorbent Assay

The levels of IL-4, IL-6, TNF-*α*, p53, and p16^INK4a^ in plasma were detected by an enzyme-linked immunosorbent assay (ELISA) according to the manufacturer's instructions.

#### 2.1.5. Fluorescence In Situ Hybridization (FISH) and *γ*-H2AX Immunofluorescence-Telomere FISH (IF-FISH)

Fluorescence in situ hybridization (FISH) of telomeres was performed on methanol/acetic acid-fixed bone marrow cells and paraffin-embedded sectioned testis tissue as previously described [30] using a Cy3-conjugated PNA telomere oligonucleotide probe (Panagene, Daejeon, KR). *γ*-H2AX immunofluorescence-telomere FISH was carried out as described by Kasbek and colleagues [31]. FISH was performed as previously described, and immunofluorescence was performed using anti-phospho-histone H2AX primary antibody (Ser 139); endogenous peroxides of testisspecimens were blocked with goat serum followed by deparaffinization and rehydration, and antigen retrieval was performed by heating in a microwave. The primary antibody and the Alexa Fluor® 488-conjugated secondary antibody were both diluted 1 : 500. Stained cells were photographed using an Olympus inverted microscope or confocal microscope (Olympus FV1000, JP).

#### 2.1.6. cDNA Library Preparation and Sequencing

cDNA libraries of testis cells were constructed and sequenced at the Novogene Bioinformatics Institute (BJ, CN). Clean data were obtained after filtering out reads with adaptors and poly-N sequences > 10% and low-quality reads from raw data through in-house Perl scripts. The Q20, Q30, and GC content of the clean reads were calculated. All downstream analyses were based on the good-quality clean reads. Differentially expressed genes (DEGs) with ∣log_2_ (fold change)∣ > 0 and *P* values < 0.05 were considered significant. In order to unveil the pathways that may be associated with the identified DEGs, KEGG pathway enrichment analysis was performed using the NovoMagic v3.0 website (https://magic.novogene.com/) (Supplementary Tables [Supplementary-material supplementary-material-1] and [Supplementary-material supplementary-material-1]).

#### 2.1.7. Quantitative Real-Time Polymerase Chain Reaction (qPCR)

The RNA-sequencing results were validated by quantitative real-time polymerase chain reaction (qPCR). Total RNA of the testis tissues was extracted using a TRIzol reagent (Invitrogen, NY, USA), and 1 *μ*g of RNA was reverse-transcribed using HiScript II Q Select RT SuperMix for qPCR (Vazyme Biotech, JS, CN) according to the manufacturer's instructions. To evaluate the expression of the involved genes, quantitative real-time PCR detection was performed with AceQ qPCR SYBR Green Master Mix (Vazyme Biotech) on the Rotor-Gene Q 2000 real-time PCR system (Qiagen, Düsseldorf, DE). All of the specific primers were synthesized by GENEWIZ, Inc. (JS, CN), and the sequences of each primer are listed in Supplementary [Supplementary-material supplementary-material-1]. Each sample was tested and analyzed in triplicate. GAPDH was used as the endogenous control.

#### 2.1.8. Assessment of Sperm Motility

The sperm motility assessment of all mice was performed as previously described [32]. One testis from each animal was placed into a 35 mm dish containing 1.5 mL of normal saline and ruptured using syringes with 26 G needles. Then, the sperms were gently squeezed out of the testes. Dishes containing released sperm were incubated at 37°C for 30 min in a 5% CO_2_ air incubator, followed by removal of the testes, mixing of the sperm suspension by gentle swirling, and subsequent assessment of sperm motility. Sperm suspensions were diluted 1 : 10 in normal saline (37°C), loaded onto a prewarmed glass slide (37°C), covered with a prewarmed glass coverslip, and rested for 20 s before analysis. Sperm motility was blind-assessed at 37°C using a microscope at ×200 magnification. At least 200 sperms from five fields were counted in each sperm sample within 2 min of removal from the incubator.

### 2.2. Human Research

#### 2.2.1. Participants and Samples

This study was carried out in accordance with the approved guidelines of the Committees for Ethical Review of Research Involving Human Subjects at Tianjin Medical University and Tianjin Worker's Hospital. Informed consent was obtained from all participants.

Participants were recruited from Tianjin Worker's Hospital. In total, 96 participants (41 males and 55 females) underwent the initial screening. All participants filled out the Pittsburgh Sleep Quality Inventory (PSQI) [33] to assess their sleep quality in the month preceding the start of the experiment, in which nineteen individual items generate seven “component” scores: subjective sleep quality, sleep latency, sleep duration, habitual sleep efficiency, sleep disturbances, use of sleeping medication, and daytime dysfunction. The sum of the scores for the seven yields the PSQI score. Good sleepers were the individuals whose PSQI scores were between 0 and 9, and the “poor” sleepers were those whose PSQI scores were between 10 and 21 (depressed patients were excluded). One portion of the participants (*N* = 63) showed good sleep quality (scores less than 9), while the rest of the participants (*N* = 33) were in the poor sleep quality range (scores between 10 and 21). As described previously, the normal and low reference values for folate was at >7.2 ng/mL and <7.2 ng/mL [22], so each group was divided into two subgroups according to the folate concentration: 7.2 ng/mL (<16.4 nmol/L) and >7.2 ng/mL (>16.4 nmol/L); thus, all participants were divided into 4 subgroups: good sleep with lower folate (GS+LF) (*N* = 26), poor sleep with lower folate (PS+LF) (*N* = 21), good sleep with higher folate (GS+HF) (*N* = 37), and poor sleep with higher folate (PS+HF) (*N* = 12). Demographic characteristics of the study population are shown in Supplementary Tables [Supplementary-material supplementary-material-1].

All samples were used anonymously. Peripheral blood was collected from each participant into ethylenediaminetetraacetic acid anticoagulant tubes and heparin-anticoagulant tubes.

#### 2.2.2. Routine Blood Examination and Telomere FISH of Leukocytes

Routine blood examination and serum folate levels were detected. The telomere length of the leukocytes was determined by FISH.

### 2.3. Statistical Analysis

Data are presented as the mean ± standard error of the mean (mean ± SEM). Statistical analysis was performed using SPSS 20.0 software (SPSS Inc., Chicago, USA). Two-way ANOVA was used for comparisons among groups, and a post hoc contrast by least significant difference (LSD) *t*-test was applied to confirm the significance. Two-tailed *P* values < 0.05 were considered statistically significant.

## 3. Results

### 3.1. Folic Acid Attenuates Sleep Deprivation-Induced Oxidative Stress and SASP

Somnipathy is a common disease and a concomitant symptom with certain diseases. It has been reported that high-grade somnipathy induces severe disorders, including circadian rhythm disorders, endocrine dyscrasia, and systemic inflammation [12, 34]. Additionally, folic acid is reported to play an important role in preventing neural tube defects and cardiovascular disease and reducing inflammation through its role as a one-carbon donor, which suggests that folic acid supplementation might benefit individuals with sleep deprivation-related disorders. To further investigate the effect of sleep deprivation on aging-related disease and the reversal effect of folic acid, a mouse model was established using the multiple classical platform technique [28, 29] for 7 days (wide platform control group (WC) *vs.* sleep deprivation group (SD)), and mice were sleep deprived for 20 h per day from 1:00 pm to 9:00 am the following day (for the experiment design, see Figure 1(a)). Before sleep deprivation, the mice were fed a folic acid-deficient diet (FAD) (*N* = 14) or an 8 mg/kg folic acid-supplemented diet (FAS) (*N* = 14) for 78 days. The serum folic acid concentration was tracked during the process. We observed that the folic acid concentration level was high (~200 ng/mL for total mice) before the customized food, since the ordinary mouse diet has relatively high folic content (4~8 mg/kg) (Figure 1(b)). As predicted, the folate concentration of the mice fed different folic acid diets showed dramatic differences (<20 ng/mL for the FAD group and >180 ng/mL for the FAS group, Figure 1(b)). Interestingly, sleep deprivation was found to lead to increased physiological dysfunctions, including ruffled fur, anemia, and mobility retardation; folic acid supplementation greatly reversed these disorders (data not shown). Additionally, the body weight was tracked every day during the folic acid feeding and sleep deprivation process. The results showed that folic acid supplementation had no effect on body weight in non-sleep-deprived mice (WC+FAD and WC+FAS) (*N* = 7 per group). However, sleep deprivation induced visible weight loss, which was abated in folic acid-fed mice (Supplementary [Supplementary-material supplementary-material-1]) (SD+FAD *vs.* SD+FAS) (*N* = 7 per group), suggesting that folic acid supplementation may help to resolve the sleep deprivation-induced physical effects.

Oxidative stress is caused by an imbalance between prooxidants and antioxidants in cells and tissues [14]. Proteins and lipids are oxidatively modified under oxidative stress and then lose/change their cellular functions [35]. It has been reported that wakefulness requires a great amount of oxygen, resulting in a significant leakage and accumulation of reactive oxygen species (ROS), such as the superoxide anion radical (O_2_^·-^), hydroxyl radicals (^·^OH), and the nonradical hydrogen peroxide (H_2_O_2_), from mitochondria during oxidative phosphorylation [35, 36]. Superoxide dismutase (SOD), catalase (CAT), and glutathione peroxidase (GPx) are the three major enzymes in charge of transforming free radicals into more stable chemical forms [35]. To determine the effect of folic acid on oxidative stress induced by sleep deprivation, we assessed the ROS level and antioxidant SOD activity in liver tissues that contain large amounts of mitochondria and are actively engaged in energy metabolism. As expected, the level of ROS was increased in the sleep deprivation group (SD+FAD *vs.* WC+FAD, SD+FAS *vs.* WC+FAS) (*N* = 7 per group), which could then be reduced by folic acid supplementation (Figure 1(c)) (SD+FAS *vs.* SD+FAD). In contrast, SOD, which specifically converts superoxide radicals to hydrogen peroxide, had the opposite change (Figure 1(d)). For instance, SOD activity was inhibited by sleep deprivation and induced by folic acid supplementation. Interestingly, we observed that folic acid supplementation could also decrease the production of ROS and promote the activity of SOD even in the non-sleep-deprived control mice (WC+FAD and WC+FAS), indicating that folic acid plays an important role in systemically mediating the redox status *in vivo*. Similarly, the antioxidative effect was also observed in testis cells (another tissue abundant with mitochondria) (Supplementary [Supplementary-material supplementary-material-1]). However, the fold change of both ROS and SOD by folic acid between the FAD group ((SD+FAD)/(WC+FAD)) and the FAS group ((SD+FAS)/(WC+FAS)) did not show visible differences, suggesting that the antioxidative function of folic acid is not specific to the sleep-deprived mice (data not shown).

Elevated ROS simulate cellular senescence and induce the alternative SASP [37]. SASP is a characteristic feature of senescent cells that secrete a predictable profile of cytokines, chemokines, and proteases. In this study, to evaluate SASP alterations, the concentration of SASP biomarkers, including IL-4, IL-6, and TNF-*α* in blood samples was measured by ELISA (*N* = 7 per group). Compared to that of the control (WC+FAD) group, the secretion of IL-4, IL-6, and TNF-*α* cytokines in the blood was increased in the sleep deprivation (SD+FAD) group of mice fed the no folic acid diet. However, the increased cytokine secretion was eliminated by folic acid supplementation (WC+FAS and SD+FAS). In contrast to the effect of folic acid on redox status, folic acid supplementation had little effect on the secretion of cytokines in control mice without sleep disturbance (WC+FAD *vs.* WC+FAS) (Figures 1(e) and 1(g); IL-4 data not shown). However, we compared the effect of folic acid between the sleep deprivation group and the control group and calculated the fold change in cytokine secretion. We observed that the secretion of TNF-*α* was dramatically reduced by the addition of folic acid (1.583 ± 0.176-fold for (SD+FAD)/(WC+FAD) *vs.*1.092 ± 0.094-fold for (SD+FAS)/(WC+FAS), *P* < 0.05) (Figure 1(f)), suggesting that folic acid plays an important role in SASP induction specifically in sleep-deprived mice.

The p53 and Rb signaling pathways are the two main signaling pathways for cellular senescence, which mediate the presentation of SASP. To further confirm the activation of the aging response signal pathways, the levels of the related regulators, including p53 and p16^INK4a^, were also detected by ELISA. We observed that sleep deprivation could activate both the p53 and Rb (p16^INK4a^) pathways (Figures 1(h)–1(k)), which may result from the increased level of ROS. Additionally, consistently, the increase of both p53 and Rb (p16^INK4a^) induced by sleep deprivation was suppressed by folic acid supplementation (1.583 ± 0.176-fold for (SD+FAD)/(WC+FAD) *vs.*1.092 ± 0.094-fold for (SD+FAS)/(WC+FAS), *P* < 0.05) (Figures 1(i) and 1(k)). Nuclear factor NF-*κ*B plays an important role in SASP signaling pathway due to its induction expression of proinflammatory factors including cytokines, chemokines, and adhesion molecules. Thus, we detected the expression of NF-*κ*B in testis sections by immunohistochemistry staining using NF-*κ*B antibody; however, we did not observe any significant change between different groups (Supplementary [Supplementary-material supplementary-material-1]). The results aforementioned showed that the secretion of the SASP biomarkers may vary between solid tissues and the circulatory system. Taken together, our data show that folic acid attenuates the sleep deprivation-induced aging response through inflammatory cytokines and aging-related protein secretion but not through the direct regulation of redox status regulation.

### 3.2. Telomere Dysfunction Could Be Ameliorated by Folic Acid Supplementation

Due to their high guanine content, telomeres are considered fragile sites of the genome and can be attacked by ROS, chemistry, ionizing radiation (IR), etc. [38, 39], and the dysfunction of telomeres leads to genomic instability and cellular senescence [40]. To investigate whether telomere function is affected by sleep deprivation and folic acid supplementation, the telomere length was determined by quantitative fluorescence in situ hybridization (Q-FISH), and telomere-specific DNA damage was determined by colocalization of *γ*-H2AX and telomeres.

**Figure 2:**
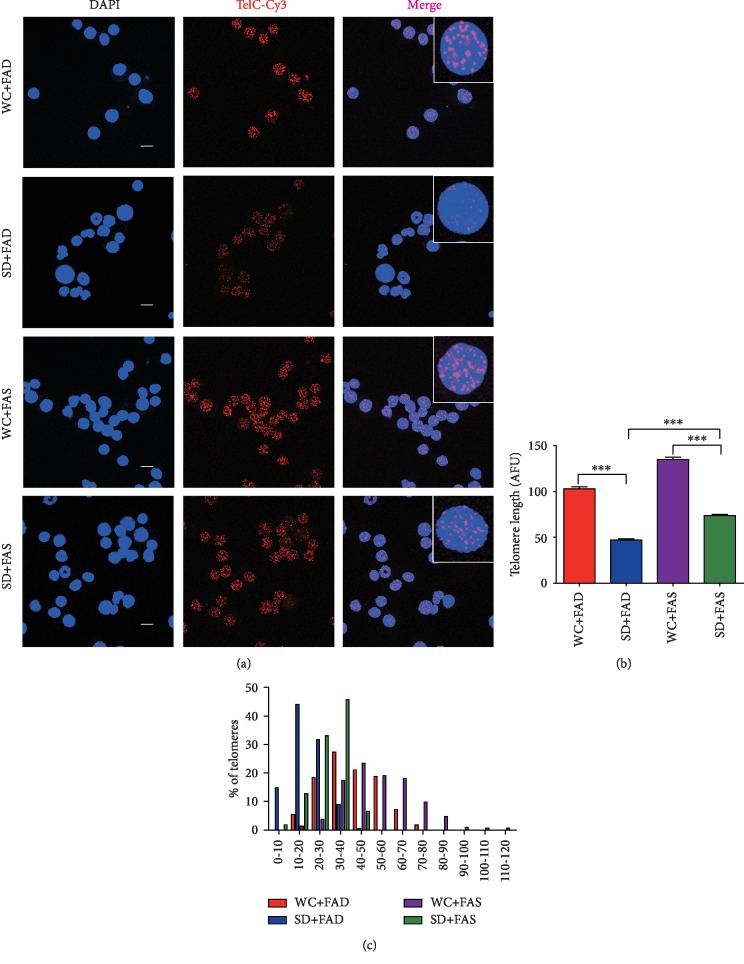
Folic acid restrains telomere shortening induced by sleep deprivation. (a) The telomere length of bone marrow cells was detected by quantitative fluorescence in situ hybridization (Q-FISH) and is shown as average fluorescence units (AFUs). Scale bars are 10 *μ*m. (b) Scatter plot of telomere AFUs for more than 200 cells of all mice. (c) The histogram displays the distribution of relative telomere length as AFUs. Data are presented as the mean ± SEM (*N* = 7 per group). ^∗∗∗^*P* < 0.001.

For telomere length detection, telomeric DNA was labeled by the Cy3 conjugated PNA probe, and the average telomere length was reflected by the fluorescent signal intensity in bone marrow cells. The data revealed that abnormal sleep rhythm led to a shortening of the average telomere length, which could be partially rescued by folic acid supplementation (Figures 2(a) and 2(b)). The telomere length (fluorescent signal intensity) distribution analysis revealed that the distribution peak moved to the left in the sleep deprivation sample. Moreover, a large number of cells (~15%) with super short telomeres (AFUs 0-10) were detected in the sleep-deprived folic acid-deficient group, suggesting that abnormal sleep rhythm may lead to telomere damage or losses other than telomere shortening (Figure 2(c)). To our surprise, compared to the SD+FAD mice, folic acid-supplemented mice (SD+FAS) had a marked recovery of the telomere loss phenotype, and the percentage of short telomeres decreased from 15% to 2% (Figure 2(c)). Additionally, this rescue effect on the non-sleep-deprived mice was clear (5.38% to 1.36%), suggesting that folic acid supplementation resolves sleep deprivation-induced telomere shortening/damage by promoting telomere DNA repair, which might be related to the suppression of p53 or other unknown causes.

**Figure 3:**
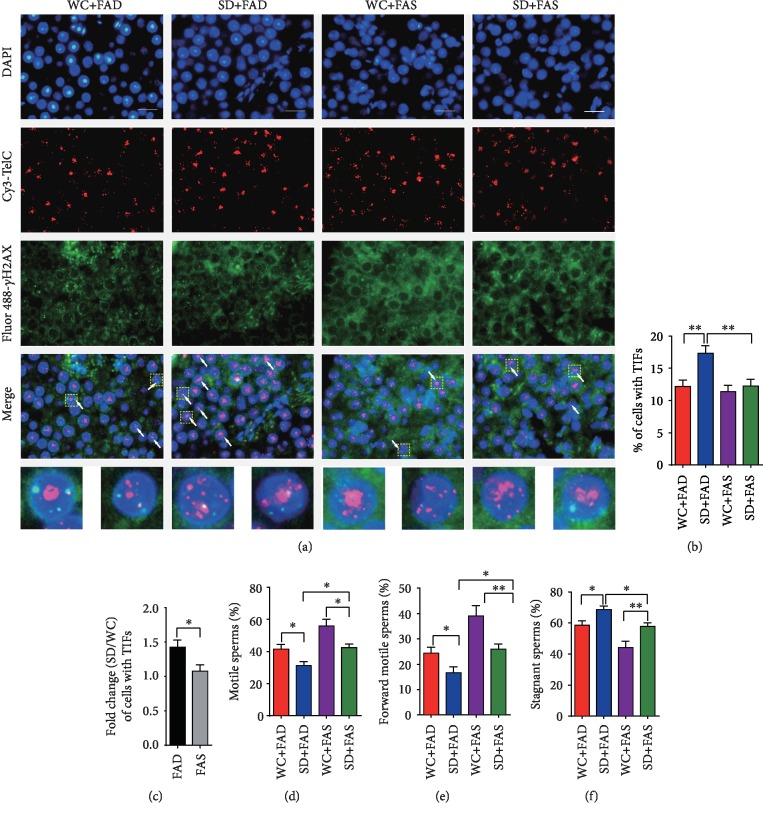
Folic acid ameliorates telomere dysfunction and rescues spermatozoa motility defects induced by sleep deprivation. (a) Telomere damage shown by the colocalization of the telomere signals and DNA damage marker *γ*-H2AX (TIFs). Scale bars are 10 *μ*m. (b, c) Representative TIFs of telomere damage images of all groups and the fold change (SD/WC) in the FAD or FAS groups. (d) Percentage of motile sperm. (e) Percentage of the forward motile sperm. (f) Percentage of stagnant sperm. Data are presented as the mean ± SEM (*N* = 7 per group). ^∗^*P* < 0.05 and ^∗∗^*P* < 0.01.

To further understand the toxic effect of sleep deprivation and folic acid on reproduction, the telomere length and telomere DNA damage-induced foci were analyzed in testis tissues; the mobility/activity of sperm was also determined. Similar to the bone marrow cell results, the loss of telomeric DNA was found to be greatly resolved by folic acid supplementation (Supplementary Figures [Supplementary-material supplementary-material-1]–[Supplementary-material supplementary-material-1]). Furthermore, the immunofluorescence colocalization data demonstrated that sleep deprivation-induced DNA damage occurred on both telomeres and other sequences of the genome. However, folic acid supplementation reduced the number of telomere dysfunction-induced foci (TIFs), and the fold change in TIFs after sleep deprivation was significantly decreased in the FAS group (1.076 ± 0.090) compared to that of the FAD group (1.426 ± 0.100) (*P* < 0.05) (Figure 3(c)), suggesting that folic acid supplementation improves telomere damage to a great extent. The number of cells with TIFs did not change when the mice with normal sleep rhythms were fed folic acid-supplemented diets. Telomere shortening or telomere damage has been suggested to play a triggering role in asthenospermia; thus, the sperm performance of mice was analyzed. Our results revealed that the percentage of active sperm, especially the forward motile sperm, was significantly decreased after sleep deprivation (24% to 16%) but increased after folic acid supplementation (16% to 26%) (Figures 3(d) and 3(e)). In addition, we also found that mice suffering from sleep deprivation had more inactive sperms (59% to 69%), which could also be rescued by folic acid supplementation (69% to 58%) (Figure 3(f)). To our surprise, folic acid supplementation also improved the sperm motility performance in mice with normal sleep rhythms, suggesting that folic acid rescues sleep deprivation-induced asthenospermia through its telomere protection function and at least one another pathway.

### 3.3. Folic Acid Recovered the Transcriptome Altered by Sleep Deprivation

**Figure 4:**
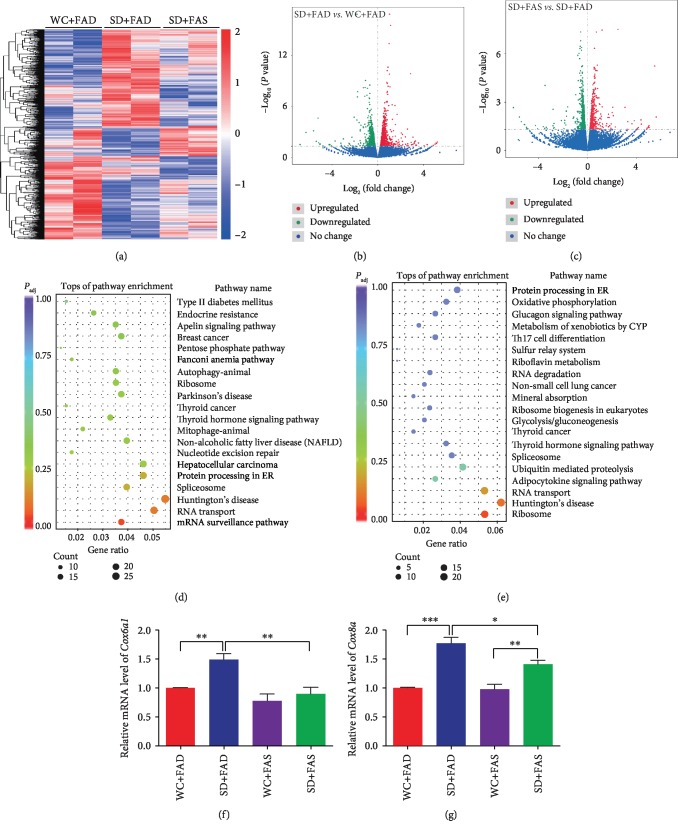
Transcriptome profile analysis. (a) Heat map of gene expression in WC+FAD, SD+FAD, and SD+FAS mice (*N* = 2 per group). (b) Volcano plot showing the transcript levels differentially expressed between SD+FAD and WC+FAD mice. (c) Volcano plot showing the transcript levels differentially expressed between SD+FAS and SD+FAD mice. (d) Top representative pathways of enriched DEGs in the SD+FAD *vs.* WC+FAD group. (e) Top representative pathways of enriched DEGs in the SD+FAS *vs.* SD+FAD group. (f) Relative expression of *Cox6a1* detected by real-time PCR. (g) Relative expression of *Cox8a* detected by real-time PCR. Data are presented as the mean ± SEM (*N* = 7 per group). ^∗^*P* < 0.05; ^∗∗^*P* < 0.01; and ^∗∗∗^*P* < 0.001.

To understand the mechanism by which folic acid effectively ameliorates the negative physical response caused by sleep deprivation, we performed RNA-sequencing (RNA-seq) to identify differentially expressed genes (DEGs) that play key roles in this process. Heat map and volcano plot analysis showed the DEGs in WC+FAD, SD+FAD, and SD+FAS mice (2 of each) (Figures 4(a)–4(c)). A total of 1667 DEGs, including 810 upregulated and 857 downregulated, were found in SD+FAD mice compared with WC+FAD mice. In contrast, 496 upregulated and 692 downregulated genes were found in SD+FAS mice compared with SD+FAD mice. The DEGs upregulated in SD+FAD mice but downregulated in SD+FAS or SD+FAD mice overlapped. The Kyoto Encyclopedia of Genes and Genomes (KEGG) analysis demonstrated that these DEGs are significantly associated with cellular metabolism and oxidative phosphorylation-related pathways (i.e., oxidative phosphorylation pathway, metabolism of xenobiotics by cytochrome P450 pathway, thyroid hormone signaling pathway, protein processing in endoplasmic reticulum, and telomere length regulation) (Figures 4(d) and 4(e); Supplementary Tables [Supplementary-material supplementary-material-1] and [Supplementary-material supplementary-material-1]). Four DEGs (*Cox6a1*, *Cox8a*, *Ndufa12*, and *Ndufb8*) related to the oxidative phosphorylation pathway were selected for further analysis, and the level of transcription was determined (Figures 4(f) and 4(g); Supplementary [Supplementary-material supplementary-material-1]). As expected, the expression of the genes was elevated by sleep deprivation but inhibited after folic acid supplementation, which illustrates that sleep deprivation plays an important role in promoting the related cellular metabolism and oxidative phosphorylation, which could be subsequently suppressed by folic acid. Taken together, our RNA-seq data show that in addition to protecting telomeres, folic acid resolves sleep deprivation-induced disorder by suppressing the oxidative phosphorylation process. Additionally, a differentially expressed gene named *Tep1*, which encodes a component of the telomerase-associated protein responsible for telomerase activity, was identified by RNA-seq. The transcription of *Tep1* was suppressed by sleep deprivation but elevated in folic acid supplementation samples. Tep1 has been reported to play an essential role in telomere length regulation, which might be the reason for the telomere elongation observed in folic acid-supplemented mice.

### 3.4. Elongated Leukocyte Telomere Length Is Associated with a High Serum Concentration Level of Folic Acid in Humans

**Figure 5:**
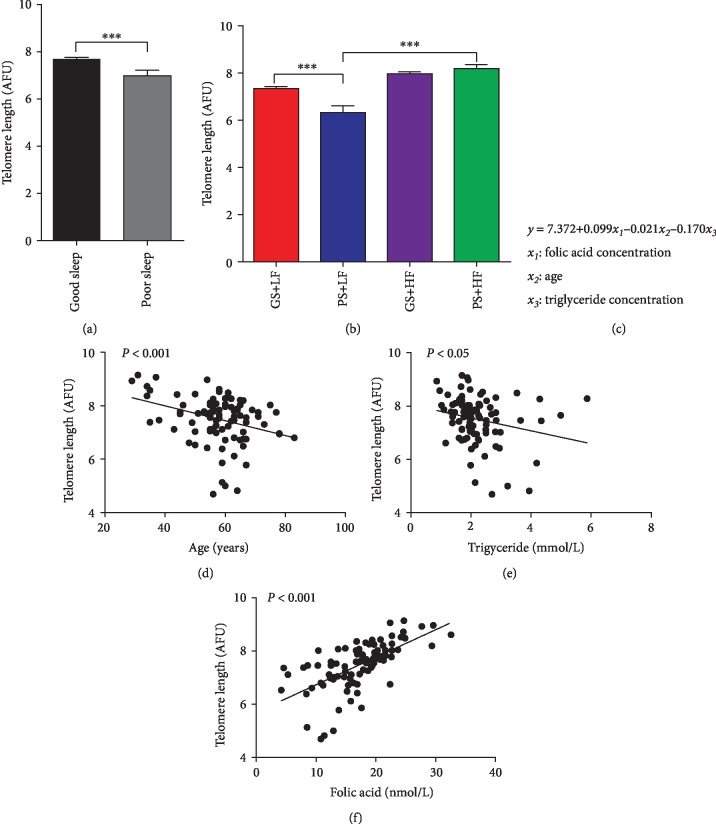
Elongated leukocyte telomere length is associated with a high concentration level of plasma folic acid in humans. (a) The leukocyte telomere length of the participants divided into two groups according to the sleep score. (b) The leukocyte telomere length of the participants divided into four groups according to both sleep score and blood folate concentration. (c) A multiple regression analysis was performed for all the individuals. (d) The correlation between leukocyte telomere length and age. (e) The correlation between leukocyte telomere length and triglycerides. (f) The correlation between leukocyte telomere length and blood folate concentration. Data are presented as the mean ± SEM. ^∗∗∗^*P* < 0.001.

Telomeres are shortened with cell division, and telomere length is considered a marker of biological age [41–43]. In our mouse experiments, we observed that folic acid supplementation could greatly ameliorate sleep deprivation-induced telomere damage/shortening. To investigate the telomere effect of sleep quality and folic acid supplementation in the human population, leukocyte telomere length was assessed by Q-FISH. A total of 98 individuals were included, and 2 were excluded due to incomplete information (detailed information is shown in Supplementary Tables [Supplementary-material supplementary-material-1]). The sleep quality of the participants was determined by the PSQI score. Two-thirds of the participants (*N* = 63) showed good sleep quality (scores less than 9), while the rest of the participants (*N* = 33) were in the poor sleep quality range (scores between 10 and 21). The individuals were divided into four groups according to the sleep score and blood folate concentration. Overall, the poor sleep group showed a shorter telomere length compared to that of the good sleep group (Figure 5(a)). The clinical parameters, including age, body mass index (BMI), folate concentration, and the routine blood indices, were put in a multiple regression equation (Figure 5(c)), and the *β* value was generated to analyze the association of these variables with telomere length. Our data demonstrated that the leukocyte telomere length of the population was significantly inversely associated with age and triglyceride concentration and positively correlated with blood folate (Figures 5(d)–5(f); Supplementary [Supplementary-material supplementary-material-1]). The population with lower levels of serum folate (PS+LF) showed weaker telomere fluorescence compared to the population with good sleep and lower serum folate levels (GS+LF). However, in contrast, the telomere length of the GS+HF group and the PS+HF group showed no significant change. Moreover, both groups with higher folate levels (GS+HF group and PS+HF group) had longer telomeres than the groups with lower folate levels (GS+LF group and PS+LF group) (Figure 5(b)). Consistent with our mouse results, our human population-based study indicates that poor sleep quantity could significantly shorten telomere length or damage telomeres, which may ultimately contribute to aging- and aging-related diseases; a high baseline concentration of folate in blood could greatly reverse the effects of sleep deficiency.

## 4. Discussion

Sleep deprivation has been reported to be associated with various human pathologies, including inflammation, cardiovascular disease, Alzheimer's disease, reproductive system dysfunction, other aging-related issues, and even death [5, 44]. The most acceptable hypothesis for these observations is that the imbalance between prooxidants and antioxidants trigged by wakefulness may play an essential role in contributing to the associated disorders [45]. However, an effective treatment method for sleep deficiency-induced disorder has not yet been developed. In our study, we observed that sleep deprivation could lead to free radical accumulation and corresponding systemic damage, as previously reported [45]. Interestingly, our data revealed that sleep deprivation induces serious telomere dysfunction and a subsequent SASP. Further investigation in the human population demonstrated that poor sleep quality led to severe telomere shortening. More importantly, we showed for the first time that folic acid supplementation could reverse telomere disorder and the secretion of senescence-associated cytokines in both mice and humans.

Folate, also known as vitamin B9, is an essential compound involved in many important biochemical processes [46]. Folate is essential for the body to make DNA, RNA, and amino acids during periods of rapid growth, such as infancy and pregnancy [47]. Recent studies have provided evidence showing that folate plays an important role in preventing NTD, cardiovascular disease, and stroke, which are diseases related to oxidative stress [24]. Since folate acts on the methionine metabolic cycle, being related to the endogenous antioxidants glutathione (GSH) and glutathione peroxidase, the most likely explanation for our findings is that folic acid could resolve the sleep deprivation-associated disturbances through its antioxidant activity. Consistently, we observed that the accumulation of ROS could be abrogated and that the activity of SOD could be induced by the addition of folic acid. However, the fold change of ROS and SOD was the same between sleep-deprived mice and non-sleep-deprived control mice after folic acid supplementation, suggesting that the antioxidation activity of folic acid is not specific to the wakefulness of mice. The subsequent results showed that folic acid treatment inhibited the secretion of senescence-associated cytokines (especially TNF-*α*) and telomere shortening/damage only for the sleep-deprived mice. To our knowledge, this is the first study showing that folic acid could help resolve the sleep deprivation-induced aging-related phenotype change and could thus provide a major advance in the understanding of the function of folic acid.

In addition, folic acid and its intermediate metabolite tetrahydrofolic acid play an essential role in the synthesis of S-adenosylmethionine (SAM), which functions as a cofactor and methyl group donor for DNA methylation [48]. We speculate that folic acid may protect cells via an epigenetic modification. Folic acid could inhibit the stress response process and aging-related inflammatory response by mediating the expression of genes involved in regulating SASP or oxidative stress, such as p53 and Rb, through methylation or demethylation of the promoter. Our RNA-seq data showed that the expression levels of the genes involved in oxidative phosphorylation were greatly altered by folic acid supplementation.

Telomeres which are a type of repetitive nucleotide sequence (5′-(TTAGGG)_*n*_-3′) at the ends of all chromosomes are considered common fragile sites of the genome. Telomeres are shortened with cell division, and telomere length is considered a marker of biological age [41, 42]. Due to their high guanine content, telomeres are highly sensitive to damage and shortening by several endogenous or exogenous factors, such as chronic oxidative stress, toxin damage, and radiation exposure [19, 20]. When telomeres become critically short or sufficiently damaged, the dysfunction triggers DNA damage signaling, leading to genomic instability and cell senescence [10, 40]. Thus, sleep deprivation, which is reported to trigger oxidative stress, leads to telomere-specific DNA damage and subsequent aging [49]. The tissues and cells with a high level of metabolic activity are easily to get free radical accumulation and subsequent DNA damage. Thus, the reproductive system is very sensitive to the external and internal stresses. Moreover, testis is one type of the tissues with telomerase activity. It has been reported that the telomerase played an import role in DNA damage response and damaged DNA repair. Our current data showed that sleep deprivation could lead to telomere damage not only in telomerase-negative cells but also in telomerase-positive cells. Interestingly, we observed that folic acid upregulated the expression of the telomerase-associated protein TEP1, which is essential for telomere elongation and telomere damage repair. Thus, we suspect that in addition to regulating oxidative status, folic acid plays a direct role in telomere length maintenance.

In summary, our study implicates folic acid as an effective vitamin to counteract telomere dysfunction and the associated senescence phenotype induced by sleep deprivation. Although the detailed mechanism needs to be further addressed, our findings indicate that to obtain a better treatment outcome of sleep deficiency-related disorders, the recovery of subsequent oxidative stress and related damage is greatly needed.

## Data Availability

The data used to support the findings of this study are available from the corresponding authors upon request.

## Supplementary Materials

Supplementary Materials Supplementary Figure 1: folic acid ameliorates weight loss induced by sleep deprivation. (A) Schematic photo of the sleep deprivation group. Mice were sleep deprived in a water tank containing multiple classical platforms (diameter in 3 cm). (B) Schematic photo of the control platform group. The control group mice were placed on large platforms (diameter in 6 cm). (C) Long-term weight change (*n* = 7 per group). Supplementary Figure 2: oxidative stress in testis cells. (A) Reactive oxygen species (ROS) in the testis cells was assayed using DCFH-DA as a probe (*n* = 7 per group). (B) Antioxidative capacity in the testis cells was detected by the total superoxide dismutase (SOD) assay kit with WST-8 (*n* = 7 per group). Data was represented as the mean ± SEM. ^∗^*P* < 0.05; ^∗∗^*P* < 0.01; ^∗∗∗^*P* < 0.001. Supplementary Figure 3: the expression of NF-*κ*B in testis cells. The testis sections immunohistochemically stained against NF-*κ*B of each group. Scale bars are 100 *μ*m. Supplementary Figure 4: folic acid restrains telomere shortening of testis cells induced by sleep deprivation. (A) Telomere length of testis cells was detected by quantitative fluorescence in situ hybridization (Q-FISH) and shown with average fluorescence unit (AFU). Scale bars are 10 *μ*m. (B) Scatter plot of AFU for more than 300 cells of all mice. (C) Histogram displays distribution of relative telomere length. Data was represented as the mean ± SEM (*n* = 7 per group). ^∗∗∗^*P* < 0.001. Supplementary Figure 5: relative expression of Ndufa12 and Ndufb8 detected by real-time PCR. (A) Relative mRNA level of Ndufa12 (*n* = 7 per group). (B) Relative mRNA level of Ndufb8. Data was represented as the mean ± SEM (*n* = 7 per group). ^∗^*P* < 0.05. Supplementary Table 1: composition of the experimental diets. Supplementary Table 2: the differentially expressed genes found in SD+FAD vs. WC+FAD group and related pathways identified with KEGG analysis. Supplementary Table 3: the differentially expressed genes found in SD+FAS vs. SD+FAD group and related pathways identified with KEGG analysis. Supplementary Table 4: primer sequences for real-time PCR detection. Supplementary Table 5: demographic characteristics of the study population. Clinical parameters are shown as the mean ± SD. Supplementary Table 6: demographic characteristics of the study population divided into two groups according to the sleep score. Clinical parameters are shown as the mean ± SD. Supplementary Table 7: demographic characteristics of the study population divided into four groups according to both sleep score and blood folic acid concentration. Clinical parameters are shown as the mean ± SD. Supplementary Table 8: linear regression analysis of the correlation between the leukocyte telomere length (AFU) and general characteristics.
