# Supplementary material for: Folic Acid Supplementation Suppresses Sleep Deprivation-Induced Telomere Dysfunction and Senescence-Associated Secretory Phenotype (SASP)
Source: Oxid Med Cell Longev. 2019 Dec 14;2019:4569614. doi: 10.1155/2019/4569614 (PMC6948340; doi:10.1155/2019/4569614)
Supplement: Supplementary Materials — Supplementary Figure 1: folic acid ameliorates weight loss induced by sleep deprivation. (A) Schematic photo of the sleep deprivation group. Mice were sleep deprived in a water tank containing multiple classical platforms (diameter in 3 cm). (B) Schematic photo of the control platform group. The control group mice were placed on large platforms (diameter in 6 cm). (C) Long-term weight change (n = 7 per group). Supplementary Figure 2: oxidative stress in testis cells. (A) Reactive oxygen species (ROS) in the testis cells was assayed using DCFH-DA as a probe (n = 7 per group). (B) Antioxidative capacity in the testis cells was detected by the total superoxide dismutase (SOD) assay kit with WST-8 (n = 7 per group). Data was represented as the mean ± SEM. ∗P < 0.05; ∗∗P < 0.01; ∗∗∗P < 0.001. Supplementary Figure 3: the expression of NF-κB in testis cells. The testis sections immunohistochemically stained against NF-κB of each group. Scale bars are 100 μm. Supplementary Figure 4: folic acid restrains telomere shortening of testis cells induced by sleep deprivation. (A) Telomere length of testis cells was detected by quantitative fluorescence in situ hybridization (Q-FISH) and shown with average fluorescence unit (AFU). Scale bars are 10 μm. (B) Scatter plot of AFU for more than 300 cells of all mice. (C) Histogram displays distribution of relative telomere length. Data was represented as the mean ± SEM (n = 7 per group). ∗∗∗P < 0.001. Supplementary Figure 5: relative expression of Ndufa12 and Ndufb8 detected by real-time PCR. (A) Relative mRNA level of Ndufa12 (n = 7 per group). (B) Relative mRNA level of Ndufb8. Data was represented as the mean ± SEM (n = 7 per group). ∗P < 0.05. Supplementary Table 1: composition of the experimental diets. Supplementary Table 2: the differentially expressed genes found in SD+FAD vs. WC+FAD group and related pathways identified with KEGG analysis. Supplementary Table 3: the differentially expressed genes found in SD+FAS vs. SD+FAD group [file 4569614.f1.pdf]

Supplementary Figure 1

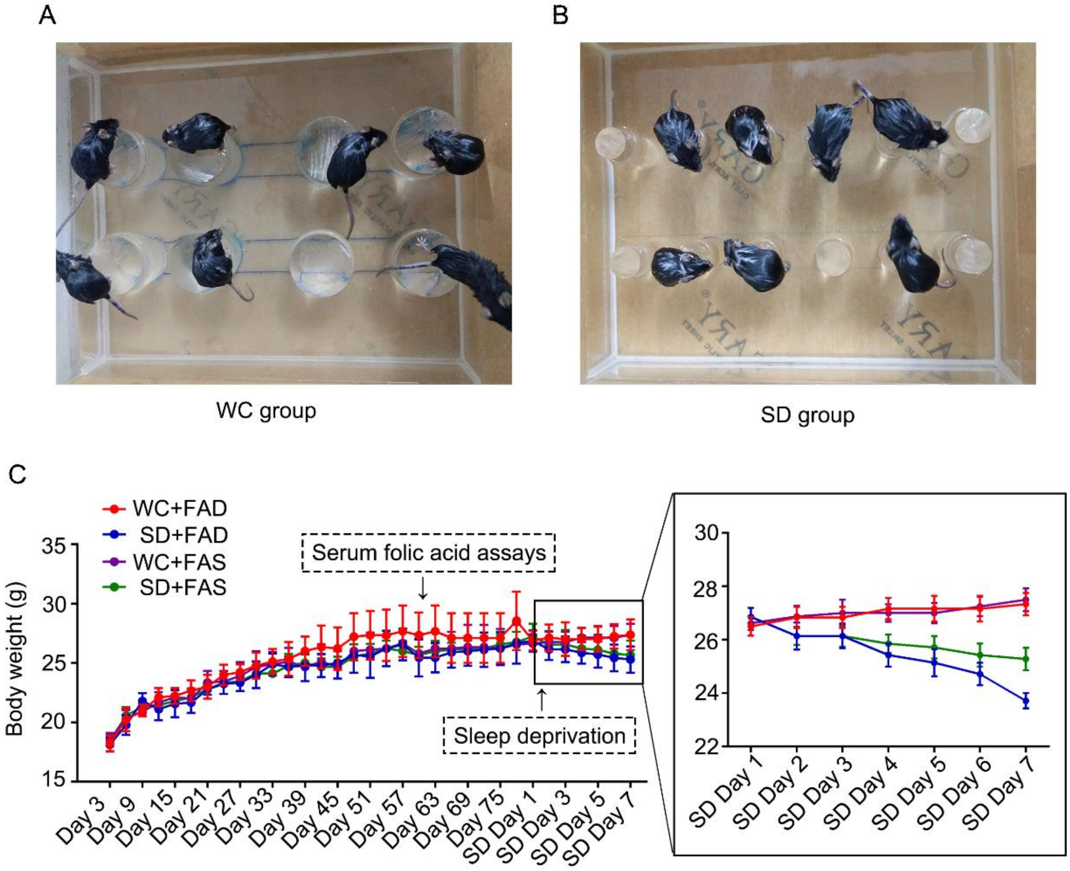

Supplementary Figure 1. Folic acid ameliorates weight loss induced by sleep deprivation. (**A**) Schematic photo of sleep deprivation group. Mice were sleep deprived in a water tank containing multiple classical platform (diameter in 3 cm). (**B**) Schematic photo of control platform group. The control group mice were placed on large platforms (diameter in 6 cm). (**C**) Long term weight change (n = 7 per group).

Supplementary Figure 2

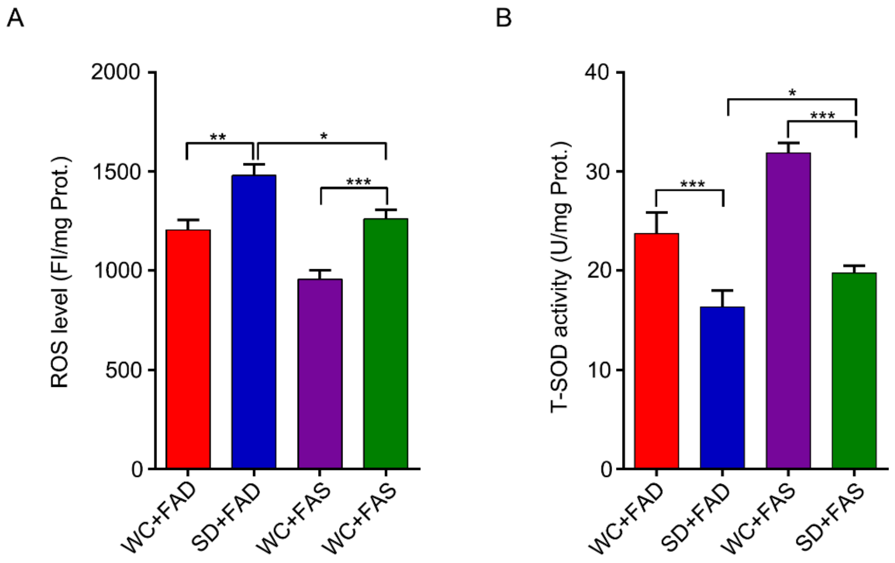

Supplementary Figure 2. The oxidative stress in testis cells. (**A**) Reactive oxygen species (ROS) in liver were assayed using DCFH-DA as a probe (n = 7 per group). (**B**) Antioxidative capacity in liver was detected by the total superoxide dismutase (SOD) assay kit with WST-8 (n = 7 per group). Data was represented as mean ± SEM. $^{*}P < 0.05$; $^{**}P < 0.01$; $^{***}P < 0.001$.

Supplementary Figure 3

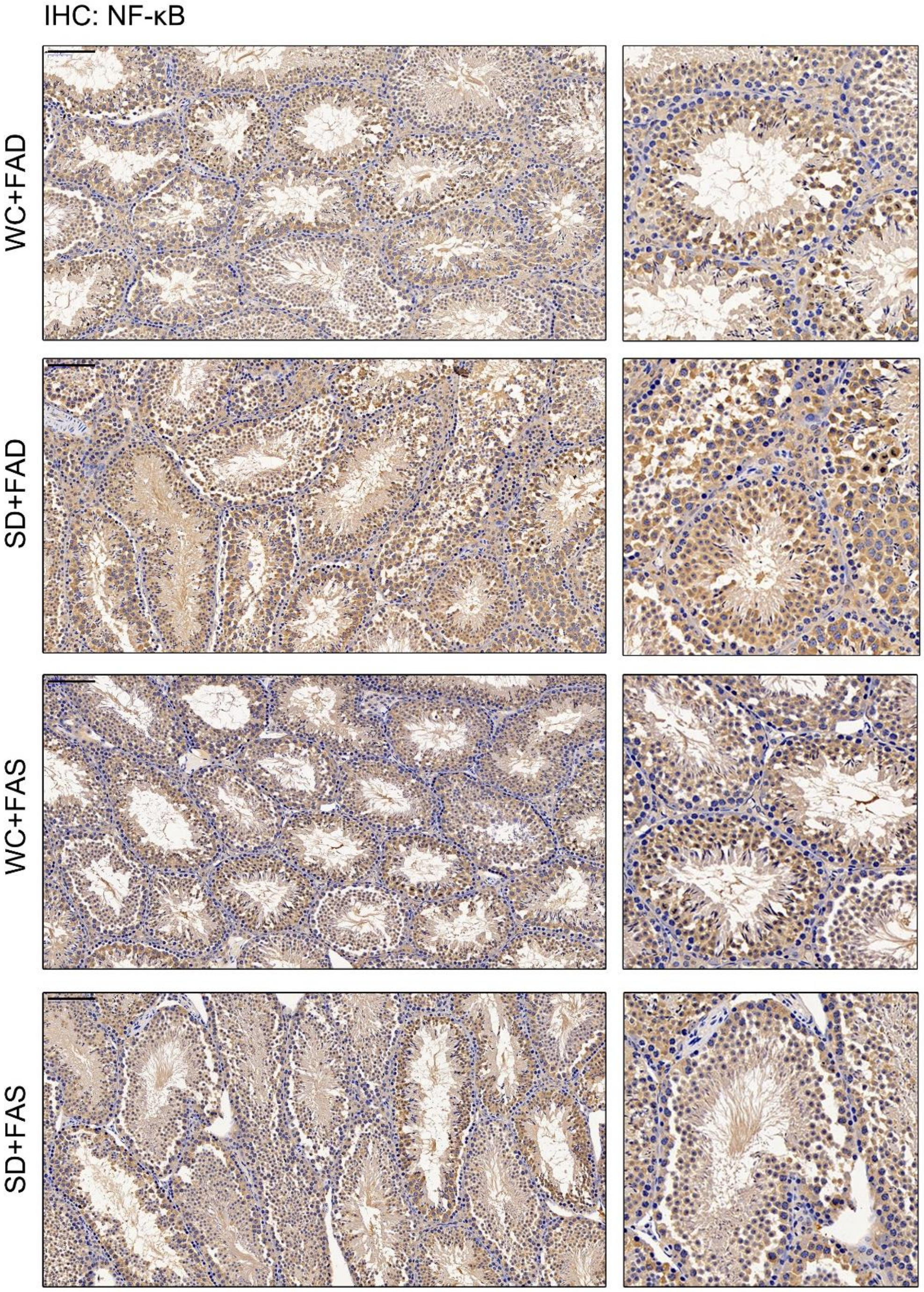

Supplementary Figure 3. The expression of NF-κB in testis cells. The testis sections immunohistochemically stained against NF-κB of each group.

Supplementary Figure 4

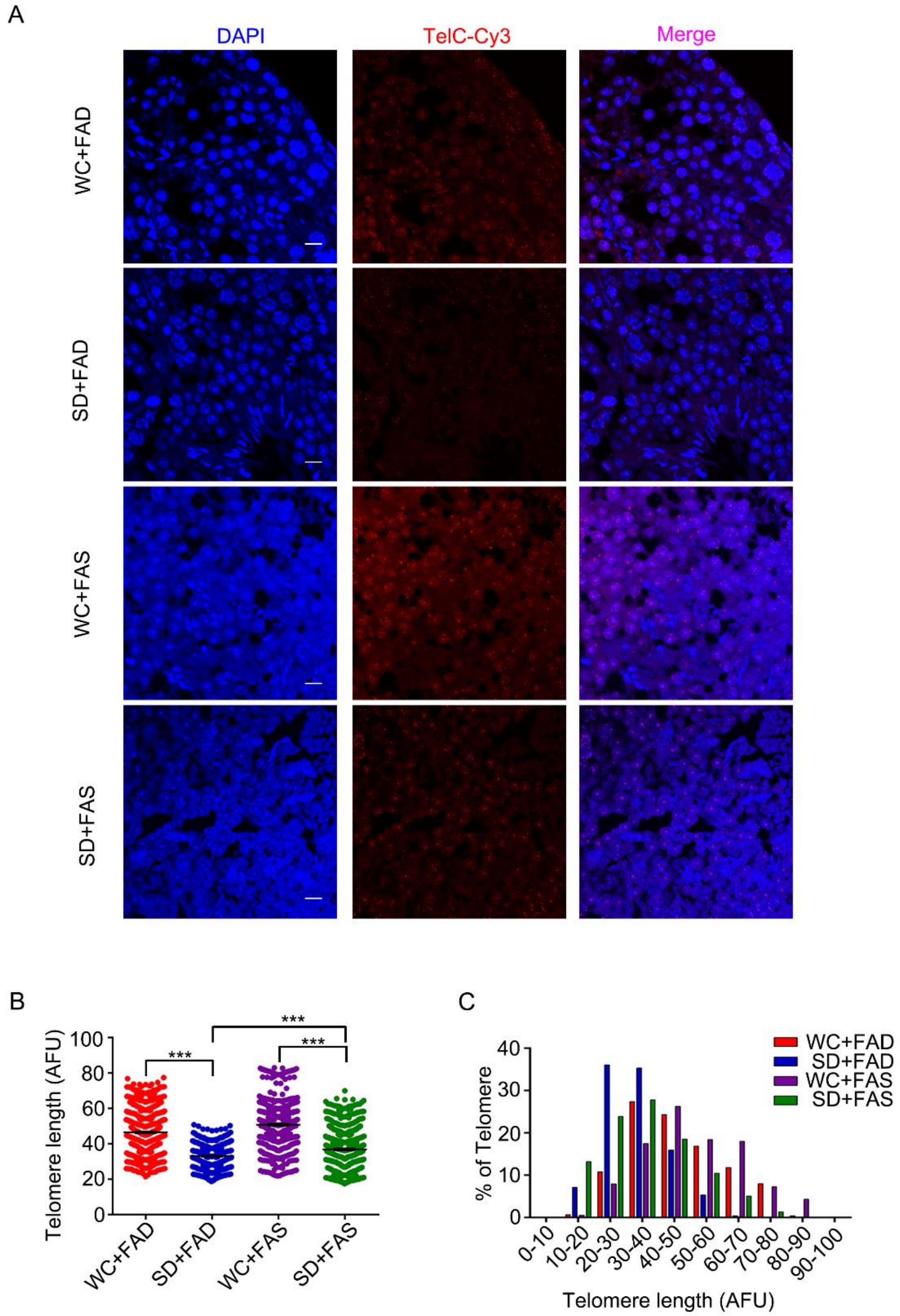

Supplementary Figure 4. Folic acid restrains telomere shorten of testis cells induced by sleep deprivation. (**A**) Telomere length of bone marrow cells was detected by quantitative fluorescence in situ hybridization (Q-FISH) and shown with average fluorescence unit (AFU) Scale bars are 10 μm. (**B**) Scatter plot of AFU for more than 300 cells of all mice. (**C**) Histogram displays distribution of relative telomere length. Data was represented as mean ± SEM (n = 7 per group). $^{***}P < 0.001$.

Supplementary Figure 5

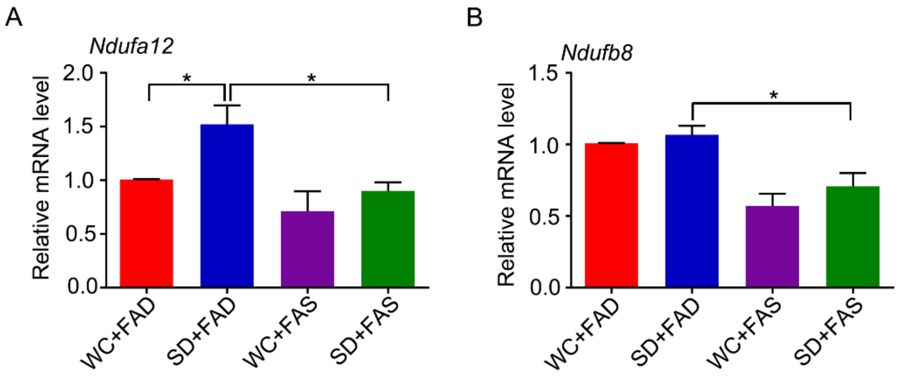

Supplementary Figure 5. Relative expression of *Ndufa12* and *Ndufb8* detected by real time PCR (**A**) Relative mRNA level of *Ndufa12* (n = 7 per group). (**B**) Relative mRNA level of *Ndufb8*. Data was represented as mean ± SEM (n = 7 per group). $^{*}P < 0.05$.

Supplementary Table 1. Composition of the experimental diets.

Ingredient	Folic acid deficiency	Folic acid supplement
Energy (kcal/g)	3.6	3.6
Protein (%)	12.5	12.5
Carbohydrate (%)	72.7	72.7
Fat (%)	4	4
Amino acid Mixture:		
Alanine (g/kg)	3.3	3.3
Arginine (g/kg)	4.5	4.5
Aspartic acid (g/kg)	8	8
Glutamate (g/kg)	25.5	25.5
Glycine (g/kg)	2.3	2.3
Lysine (g/kg)	9.2	9.2
Methionine (g/kg)	3.3	3.3
Cysteine (g/kg)	2.4	2.4
Tryptophan (g/kg)	1.6	1.6
Proline (g/kg)	14.3	14.3
Serine (g/kg)	6.7	6.7g
Histidine (g/kg)	3.3	3.3
Leucine (g/kg)	10.9	10.9
Isoleucine (g/kg)	5.9	5.9
Phenylalanine (g/kg)	6.2	6.2
Tyrosine (g/kg)	6.6	6.6
Threonine (g/kg)	4.7	4.7
Valine (g/kg)	7	7
Mineral Mixture:		
Calcium (mg/kg)	5000	5000
Phosphorus (mg/kg)	3000	3000
Potassium (mg/kg)	3600	3600
Sodium (mg/kg)	1033	1033
Magnesium (mg/kg)	511	511
Iron (mg/kg)	45	45
Zinc (mg/kg)	35	35
Manganese (mg/kg)	10	10
Copper (mg/kg)	6	6
Iodide (mg/kg)	0.2	0.2
Chromium (mg/kg)	1	1
Inorganic sulfur (mg/kg)	300	300
Chloride (mg/kg)	1613	1613
Vitamin Mixture:		
Vitamin A (IU/g)	4	4
Vitamin D (IU/g)	1	1
Vitamin E (IU/g)	0.075	0.075
Vitamin K (mg/kg)	0.86	0.86
Thiamine (mg/kg)	5	5

Riboflavin (mg/kg)	6	6
Nicotinic acid (mg/kg)	30	30
Pantothenic acid (mg/kg)	15	15
Vitamin B6 (mg/kg)	6	6
Choline (mg/kg)	1000	1000
Biotin (mg/kg)	0.2	0.2
Folate (mg/kg)	-	8

Supplementary Table 2. The differentially expressed genes found in SD+FAD *vs.* WC+FAD group and related pathways identified with KEGG analysis.

Pathways	*P* value	Genes	Count
mRNA surveillance pathway	7.99E-05	*Pabpc2, Pabpc6, Pabpn1, Upf3a, Ppp2r5c, Pabpc1, Pcf11, Pnn, Papolb, Ppp2r1b, Smg1, Ncbp2, Ppp2ca, Sympk, Cstf1, Ppp1cc, Upf2*	17
RNA transport	0.000750403	*Pabpc2, Eif5b, Eif3j2, Pabpc6, Fxr1, Upf3a, Thoc7, Pabpc1, Eif3a, Pnn, Tpr, Eif4g3, Nup210l, Eif3i, Kpnb1, Eif1ax, Eif2s1, Nupl1, Ncbp2, Nup35, Eif3j1, Upf2, Nup188*	23
Huntington's disease	0.001017	*Dnah12, Bax, Polr2a, mt-Co1, Bbc3, Ap2s1, Cox8a, Dnah7c, Ndufa11, Ndufa5, Polr2l, Dnal4, mt-Atp6, Dnah6, Atp5d, Dnaic1, Ndufc1, Uqcrc1, mt-Co2, Tfam, Sp1, Ndufab1, Cox6a1, Ndufv3, Rcor1*	25
Spliceosome	0.002796	*Prpf38b, Snrnp70, Rbmxl2, Rbm17, Hnrnpu, Snrpf, Prpf40a, Sf3a2, Sf3b4, Ncbp2, Ddx46, U2surp, Lsm5, Hspa1a, Rbm25, Snw1, Srsf5, Slu7*	18
Protein processing in endoplasmic reticulum	0.003627	*Ube2g1, Bax, Rrbp1, Erlec1, Bag1, Hspa4l, Dad1, Sec63, Fbxo2, Rad23b, Stt3a, Hspa5, Eif2s1, Ckap4, Calr, Hspa1a, Hsp90b1, Fbxo6, Sec62, Atf6, Lman2*	21
Hepatocellular carcinoma	0.00598	*Frat1, Frat2, Smarca2, Bax, Gstm1, Tgfbr1, Gstm2, Smarce1, Mtor, Pik3r2, Polk, Pten, Arid2, Smad4, Fzd1, Wnt6, Wnt4, Raf1, Gstm6, Map2k2, Mapk3*	21
Nucleotide excision repair	0.008548	*Rad23b, Pold3, Cul4b, Gtf2h1, Ercc1, Xpa, Pole3, Pold4*	8
Non-alcoholic fatty liver disease (NAFLD)	0.009802	*Bax, mt-Co1, Cox8a, Ndufa11, Ndufa5, Cyp2e1, Rxra, Pik3r2, Eif2s1, Adipoq, Ndufc1, Uqcrc1, mt-Co2, Ndufab1, Cox6a1, Srebf1, Ndufv3, Prkag1*	18
Mitophagy - animal	0.010851	*Tfeb, Fis1, Usp8, Ubb, Becn1, Usp15, Mfn2, Ulk1, Mfn1, Sp1*	10
Thyroid hormone signaling pathway	0.011074	*Atp1a4, Plcd4, Rxra, Mtor, Pik3r2, Notch4, Med1, Ncor1, Wnt4, Plcd1, Med24, Raf1, Map2k2, Mapk3, Med12l*	15
Thyroid cancer	0.012417	*Bax, Tpr, Rxra, Polk, Pax8, Map2k2, Mapk3*	7
Parkinson's disease	0.012692	*Ube2g1, mt-Co1, Cox8a, Ndufa11, Ndufa5, mt-Atp6, Ubb, Uchl1, Drd2, Atp5d, Ndufc1, Uqcrc1, mt-Co2, mt-Nd3, Ndufab1, Cox6a1, Ndufv3*	17
Ribosome	0.015344	*Gm11808, Mrpl4, Rpsa, Rplp1, Rps10, Rpl29, Rpl10, Rps27rt, Rps19, Rpl12, Rpl19, Rpl39l, Rpl13, Rps5, Rpl34, Rps13*	16
Autophagy - animal	0.015344	*Sh3glb1, Stx17, Rb1cc1, Mtor, Pik3r2, Eif2s1, Becn1, Pten, Ppp2ca, Akt1s1, Ctsd, Ulk1, Raf1, Ulk2, Map2k2, Mapk3*	16
Fanconi anemia pathway	0.020713	*Polk, Wdr48, Rev3l, Faap100, Ercc1, Atr, Usp1, Blm*	8

Pentose phosphate pathway	0.020996	*Pgls, Aldoart1, Gpi1, Tkt, G6pd2, Rgn*	6
Breast cancer	0.022733	*Frat1, Frat2, Bax, Dll3, Mtor, Pik3r2, Polk, Pten, Fzd1, Notch4, Wnt6, Wnt4, Jag1, Sp1, Raf1, Map2k2, Mapk3*	17
Apelin signaling pathway	0.022831	*Klf2, Tgfbr1, Gnb2, Adcy10, Mtor, Becn1, Smad4, Prkce, Tfam, Acta2, Jag1, Gng5, Raf1, Map2k2, Prkag1, Mapk3*	16
Endocrine resistance	0.024689	*Bax, Dll3, Mtor, Pik3r2, Notch4, Med1, Ncor1, Jag1, Sp1, Raf1, Map2k2, Mapk3*	12
Type II diabetes mellitus	0.034009	*Cacna1a, Socs1, Mtor, Pik3r2, Adipoq, Prkce, Mapk3*	7
MicroRNAs in cancer	0.034732	*Pdcd4, Pim1, Rdx, Socs1, Mtor, Cd44, Pten, Kif23, Pak4, Bmi1, Notch4, Prkce, Raf1, Map2k2, Hoxd10, Ezh2*	16
Platinum drug resistance	0.036425	*Bax, Gstm1, Bbc3, Gstm2, Pik3r2, Rev3l, Ercc1, Xpa, Gstm6, Mapk3*	10
Lysine degradation	0.045754	*Ash1l, Kmt2d, Ogdh, Kmt2e, Hadha, Prdm2, Nsd1, Ezh2*	8
Ubiquitin mediated proteolysis	0.049492	*Ube2g1, Ube2r2, Cul3, Herc2, Ube2s, Fbxw7, Ube2u, Fbxo2, Ubr5, Birc6, Socs1, Cul4b, Ube2k, Pias2, Btrc*	15
Notch signaling pathway	0.050856	*Cir1, Psenen, Dll3, Ctbp1, Notch4, Jag1, Snw1*	7
Oxidative phosphorylation	0.059754	*mt-Co1, Cox8a, Ndufa11, Ndufa5, mt-Atp6, Tcirg1, Atp5d, Ndufc1, Uqcrc1, mt-Co2, mt-Nd3, Ndufab1, Cox6a1, Ndufv3*	14
Renin-angiotensin system	0.060221	*Agt, Klk1b24, Ctsa, Klk1b21, Klk1b22*	5
Carbon metabolism	0.061449	*Phgdh, Pgls, Aldoart1, Ogdh, Gpi1, Hadha, Tkt, G6pd2, Dlat, Acss1, Mdh2, Rgn, 4933405O20Rik*	13
Endocrine and other factor-regulated calcium reabsorption	0.072198	*Atp1a4, Ap2s1, Klk1b24, Dnm2, Klk1b21, Klk1b22, Dnm1*	7
Prion diseases	0.075213	*Bax, Hspa5, Hspa1a, Map2k2, Mapk3*	5
AMPK signaling pathway	0.076056	*Scd1, Ppp2r5c, Ppp2r1b, Mtor, Pik3r2, Adipoq, Ppp2ca, Akt1s1, Elavl1, Ulk1, Srebf1, Rab10, Prkag1*	13
Th17 cell differentiation	0.076549	*Tgfbr1, H2-Aa, Il17d, Jak3, Rxra, Mtor, Nfkbib, Rara, Smad4, Ppp3ca, Mapk3*	11
Gastric cancer	0.081251	*Frat1, Frat2, Bax, Tgfbr1, Rxra, Mtor, Pik3r2, Polk, Smad4, Fzd1, Wnt6, Wnt4, Raf1, Map2k2, Mapk3*	15
Metabolism of xenobiotics by cytochrome P450	0.084591	*Akr7a5, Gstm1, Gstm2, Cyp2e1, Ephx1, Dhdh, Gstm6*	7
Non-small cell lung cancer	0.08526	*Bax, Jak3, Rxra, Pik3r2, Polk, Raf1, Map2k2, Mapk3*	8
Chronic myeloid leukemia	0.09008	*Bax, Tgfbr1, Pik3r2, Polk, Ctbp1, Smad4, Raf1, Map2k2, Mapk3*	9

Prolactin signaling pathway	0.097527	*Socs7, Lhb, Socs1, Pik3r2, Cyp17a1, Raf1, Map2k2, Mapk3*	8
Pyruvate metabolism	0.101116	*Ldhc, Hagh, Dlat, Acss1, Mdh2*	5
Sphingolipid signaling pathway	0.10152	*Bax, Ppp2r5c, Ppp2r1b, Pik3r2, Pten, Ppp2ca, Prkce, Ctsd, Raf1, Map2k2, Mapk3, Rock2*	12
RNA degradation	0.101677	*Pabpc2, Exosc4, Pabpc6, Pabpc1, Exosc5, Lsm5, Pan3, Dcp1b, Exosc3*	9
Riboflavin metabolism	0.102068	*Rfk, Blvrb*	2
Endometrial cancer	0.105313	*Bax, Pik3r2, Polk, Pten, Raf1, Map2k2, Mapk3*	7
Wnt signaling pathway	0.110899	*Frat1, Frat2, Sox17, Chd8, Nfatc4, Ctbp1, Smad4, Fzd1, Daam1, Wnt6, Wnt4, Btrc, Ppp3ca, Rock2*	14
Protein export	0.113833	*Srp72, Sec63, Hspa5, Sec62*	4
Glioma	0.117696	*Bax, Mtor, Pik3r2, Polk, Pten, Raf1, Map2k2, Mapk3*	8
PPAR signaling pathway	0.12488	*Fabp4, Scd1, Ubc, Rxra, Adipoq, Acsbg2, Dbi, Slc27a1*	8
Alzheimer's disease	0.126687	*Psenen, mt-Co1, Cox8a, Ndufa11, Ndufa5, mt-Atp6, Atp5d, Ndufc1, Uqcrc1, mt-Co2, Ndufab1, Cox6a1, Atf6, Ndufv3, Ppp3ca, Mapk3*	16
Jak-STAT signaling pathway	0.126963	*Csf2ra, Crlf2, Il17d, Socs7, Pim1, Jak3, Socs1, Mtor, Pik3r2, Pias2, Ctf1, Il3ra, Raf1*	13
Mineral absorption	0.130869	*Mt1, Atp1a4, Atox1, Mt2, Ftl1*	5
Fc gamma R-mediated phagocytosis	0.134195	*Asap1, Vasp, Arpc5l, Pik3r2, Prkce, Dnm2, Arpc2, Raf1, Mapk3*	9
Colorectal cancer	0.139916	*Bax, Tgfbr1, Bbc3, Pik3r2, Polk, Smad4, Raf1, Mapk3*	8
Central carbon metabolism in cancer	0.145291	*Mtor, Pik3r2, Pten, G6pd2, Raf1, Map2k2, Mapk3*	7
Pancreatic cancer	0.14776	*Bax, Tgfbr1, Mtor, Pik3r2, Polk, Smad4, Raf1, Mapk3*	8
mTOR signaling pathway	0.152368	*Mtor, Pik3r2, Pten, Fzd1, Wnt6, Akt1s1, Tti1, Rps6ka1, Wnt4, Ulk1, Raf1, Ulk2, Map2k2, Mapk3*	14
Apoptosis - multiple species	0.165185	*Bax, Bbc3, Birc6, Becn1*	4
Cellular senescence	0.165887	*Tgfbr1, Zfp36l1, Lin37, Mtor, Pik3r2, Nfatc4, Pten, Ccnb3, Btrc, Ppp1cc, Atr, Raf1, Ppp3ca, Map2k2, Mapk3*	15
Citrate cycle (TCA cycle)	0.179142	*Ogdh, Dlat, Mdh2, 4933405O20Rik*	4
Autophagy - other	0.179142	*Mtor, Becn1, Ppp2ca, Ulk2*	4
Acute myeloid leukemia	0.181731	*Pim1, Mtor, Pik3r2, Rara, Raf1, Map2k2, Mapk3*	7
Base excision repair	0.193477	*Pold3, Poll, Pole3, Pold4*	4
Lipoic acid metabolism	0.194338	*Lias*	1
Non-homologous end-joining	0.200944	*Poll, Polm*	2
VEGF signaling pathway	0.202041	*Hspb1, Pik3r2, Raf1, Ppp3ca, Map2k2, Mapk3*	6

Supplementary Table 3. The differentially expressed genes found in SD+FAS *vs.* SD+FAD group and related pathways identified with KEGG analysis.

Pathways	*P* value	Genes	Count
Ribosome	8.58E-05	*Rps14, Rpl9, Mrps12, Mrpl4, Rps16, Rpl15, Rpl18a, Rpl18, Rpl34, Rpsa, Fau, Rps19, Gm11808, Rpl8, Mrpl22, Rplp0, Rps2, Rps3*	18
Huntington's disease	0.000571	*Dnah2, Sdhd, Ndufb8, Dnal4, Ep300, Bbc3, Dnah12, Dnah17, Cox6a1, Dnah7c, Tfam, Htt, Cyc1, Cox5b, Polr2f, Atp5d, Dnah7b, Uqcrb, Atp5g2, Cltb, Cycs*	21
RNA transport	0.001693	*Eif3j2, Eif5b, Pabpc6, Tpr, Fxr1, Nup62, Gemin7, Pabpc5, Trnt1, Eif2s2, Eif3a, Eif3j1, Eif3b, Thoc7, Pnn, AF366264, Eif3d, Eif5*	18
Adipocytokine signaling pathway	0.009201	*Acsbg2, G6pc3, Cpt1c, Nfkbib, Rxra, Adipor1, Rxrb, Akt2, Mtor*	9
Ubiquitin mediated proteolysis	0.009879	*Ube2u, Herc2, Ube2s, Ube2g1, Cul3, Ubr5, Fzr1, Huwe1, Pias4, Cdc34, Fbxw7, Anapc11, Stub1, Cul4b*	14
Spliceosome	0.029773	*Snrpc, Xab2, Ddx46, Rbm25, Prpf38a, Prpf40a, Hnrnpu, Snrpb, Lsm4, Sf3b5, U2surp, Lsm2*	12
Thyroid hormone signaling pathway	0.030258	*Ncor1, Ep300, Map2k2, Hras, Rxra, Fxyd2, Atp1a4, Notch4, Rxrb, Akt2, Mtor*	11
Thyroid cancer	0.039433	*Tpr, Map2k2, Hras, Rxra, Rxrb*	5
Glycolysis / Gluconeogenesis	0.045001	*Tpi1, Eno1, G6pc3, Aldh3b2, Gpi1, Aldoa, Pkm*	7
Ribosome biogenesis in eukaryotes	0.046202	*Mdn1, Eif6, Rexo1, Bms1, Nhp2, Gnl2, Fbll1, Csnk2a2*	8
Mineral absorption	0.047957	*Hmox2, Fxyd2, Atp1a4, Mt2, Ftl1*	5
Non-small cell lung cancer	0.051767	*Map2k2, Hras, Rxra, Eml4, Rxrb, Akt2, Stk4*	7
RNA degradation	0.055612	*Eno1, Pabpc6, Dis3, Exosc4, Pabpc5, Exosc5, Lsm4, Lsm2*	8
Riboflavin metabolism	0.060164	*Rfk, Flad1*	2
Sulfur relay system	0.060164	*Urm1, Mocs3*	2
Th17 cell differentiation	0.060952	*Hsp90aa1, Il17d, Nfkbib, Rxra, H2-Ab1, Ppp3ca, Rora, Rxrb, Mtor*	9
Metabolism of xenobiotics by cytochrome P450	0.061831	*Gstp1, Akr7a5, Gstm2, Aldh3b2, Cbr1, Dhdh*	6
Glucagon signaling pathway	0.064232	*Phkg2, G6pc3, Ep300, Cpt1c, Ppp4r3b, Ppp3ca, Ppp4c, Akt2, Pkm*	9
Oxidative phosphorylation	0.067536	*Sdhd, Ndufb8, Atp6v1b1, Cox6a1, Cyc1, Cox5b, Atp6v0b, Atp5d, Uqcrb, Atp5g2, Atp6v0e*	11
Protein processing in endoplasmic reticulum	0.070579	*Ubxn6, Ube2g1, Hsp90aa1, Dnajb1, Hspa4l, Dad1, Sec63, Erlec1, Ddost, Atf6b, Hsp90b1, Stub1, Rrbp1*	13
Phenylalanine metabolism	0.071004	*Aldh3b2, Mif, Il4i1*	3

Apoptosis - multiple species	0.072523	*Bbc3, Ngfr, Bok, Cycs*	4
mTOR signaling pathway	0.078309	*Akt1s1, Map2k2, Clip1, Lrp5, Atp6v1b1, Slc7a5, Hras, Lamtor1, Fzd1, Dvl2, Akt2, Mtor*	12
Proteasome	0.07957	*Adrm1, Psma6, Psmb6, Psmb4, Psme2*	5
Renin-angiotensin system	0.07968	*Ace, Klk1b24, Thop1, Klk1b21*	4
Synaptic vesicle cycle	0.0866	*Atp6v1b1, Stx1a, Napa, Atp6v0b, Cltb, Atp6v0e*	6
Taurine and hypotaurine metabolism	0.09039	*Ado, Gadl1*	2
Proximal tubule bicarbonate reclamation	0.090463	*Car2, Fxyd2, Atp1a4*	3
Parkinson's disease	0.094134	*Ube2g1, Sdhd, Ndufb8, Cox6a1, Cyc1, Cox5b, Atp5d, Uqcrb, Atp5g2, Sncaip, Cycs*	11
Platinum drug resistance	0.094812	*Gstp1, Bbc3, Gstm2, Rev3l, Atp7b, Akt2, Cycs*	7
Fructose and mannose metabolism	0.103138	*Tpi1, Tsta3, Sord, Aldoa*	4
Bile secretion	0.103727	*Car2, Rxra, Fxyd2, Abcc3, Aqp9, Atp1a4*	6
Central carbon metabolism in cancer	0.103727	*Map2k2, Slc7a5, Hras, Akt2, Mtor, Pkm*	6
MicroRNAs in cancer	0.105536	*Rdx, Pim1, Ep300, Map2k2, Hras, Pdcd4, Rock1, Tnxb, Notch4, Pak4, Mtor*	11
Synthesis and degradation of ketone bodies	0.106842	*Oxct2b, Oxct2a*	2
Tyrosine metabolism	0.111587	*Aldh3b2, Mif, Il4i1, Aox4*	4
Prostate cancer	0.114783	*Gstp1, Hsp90aa1, Ep300, Map2k2, Hras, Hsp90b1, Akt2, Mtor*	8
Non-alcoholic fatty liver disease (NAFLD)	0.117713	*Sdhd, Ndufb8, Cox6a1, Rxra, Cyc1, Cox5b, Adipor1, Uqcrb, Mlxip, Akt2, Cycs*	11
Carbohydrate digestion and absorption	0.120334	*G6pc3, Fxyd2, Atp1a4, Akt2*	4
Endocrine and other factor-regulated calcium reabsorption	0.127452	*Klk1b24, Fxyd2, Klk1b21, Atp1a4, Cltb*	5
Renal cell carcinoma	0.135854	*Egln2, Ep300, Map2k2, Hras, Pak4, Akt2*	6
HIF-1 signaling pathway	0.136239	*Eno1, Egln2, Ep300, Map2k2, Tlr4, Aldoa, Akt2, Mtor*	8
Jak-STAT signaling pathway	0.141252	*Socs7, Pim1, Il17d, Ep300, Hras, Pias4, Stat4, Akt2, Mtor, Aox4*	10
Collecting duct acid secretion	0.14711	*Car2, Atp6v1b1, Atp6v0e*	3
Apoptosis	0.151277	*Map2k2, Bbc3, Hras, Tuba3a, Tuba1c, Tuba3b, Tuba4a, Spta1, Akt2, Cycs*	10

Hepatocellular carcinoma	0.155526	*Frat1, Smarca2, Gstp1, Map2k2, Lrp5, Hras, Gstm2, Fzd1, Dvl2, Arid2, Akt2, Mtor*	12
mRNA surveillance pathway	0.166097	*Pabpc6, Smg1, Ppp2r5c, Pabpc5, Pnn, Pcf11, Etf1*	7
Longevity regulating pathway	0.166097	*Akt1s1, Hras, Rb1cc1, Atf6b, Adipor1, Akt2, Mtor*	7
Lysine degradation	0.176728	*Kmt2d, Ash1l, Kmt2c, Kmt2a, Prdm2*	5
Biosynthesis of amino acids	0.179756	*Tpi1, Eno1, Mat2a, Ass1, Aldoa, Pkm*	6
Wnt signaling pathway	0.183416	*Frat1, Rock2, Sox17, Ep300, Lrp5, Ppp3ca, Fzd1, Csnk2a2, Dvl2, AF366264*	10
Longevity regulating pathway - multiple species	0.194564	*Akt1s1, Clpb, Hras, Akt2, Mtor*	5
Cardiac muscle contraction	0.195529	*Cox6a1, Fxyd2, Cyc1, Atp1a4, Cox5b, Uqcrb*	6
Galactose metabolism	0.198693	*G6pc3, Galk1, B4galt2*	3
Autophagy - animal	0.212374	*Zfyve1, Vamp8, Akt1s1, Map2k2, Sh3glb1, Hras, Rb1cc1, Akt2, Mtor*	9

Supplementary Table 4. Primer sequences for real time PCR detection.

Genes	Sense primers (5'→3')	Antisense primers (5'→3')	Product length (bp)
Ndufb8	GCCAAGAAGTATAACATG CGA	CATGTCTAGGTCCCAGTGT ATC	180
Ndufa12	AAAAACACATTCTGGGAT GTGG	GGTGGAATAAGGAACGTAT TGC	177
Cox6a1	ATGCTCAACGTGTTCCTC AAG	TTACTCATCTTCATAGCCGG TC	171
Cox8a	ATATCACCATTGGGCTCAC TTC	CTTCTTGTAGCTCTCCAGG TG	86
Gapdh	GGTTGTCTCCTGCGACTT CA	TGGTCCAGGGTTTCTTACT CC	183

Notes.

Ndufb8, NADH: ubiquinone oxidoreductase subunit B8; Ndufa12, NADH: ubiquinone oxidoreductase subunit A12; Cox6a1, cytochrome c oxidase subunit 6A1; Cox8a, cytochrome c oxidase subunit 8A; Gapdh, glyceraldehyde-3-phosphate dehydrogenase.

Supplementary Table 5. Demographic characteristics of study population. Clinical parameters are shown as Mean ± SD.

Characteristics	Total individuals (N=96)
Age (years)	57.51±10.50
BMI (kg/m^2)	23.78±3.23
Sex (Male/Female)	41/55
Smoking (Never/Yes)	61/35
Drinking (Never/Yes)	63/33
Glucose (mmol/L)	5.42±0.85
Total cholesterol (mmol/L)	5.21±0.36
Triglyceride (mmol/L)	2.19±0.83
ALT (U/L)	32.24±29.11
AST (U/L)	32.06±24.34
Uric acid (μmol/L)	341.98±88.25
Folic acid (nmol/L)	17.50±5.35
Telomere length (AFU)	7.51±0.87

Supplementary Table 6. Demographic characteristics of study population divided into two groups according to the sleep score. Clinical parameters are shown as Mean ± SD.

Characteristics	Good sleep (N=63)	Poor sleep (N=33)	*P* value
Age (years)	56.81±9.90	58.76±11.01	0.381
BMI (kg/m^2)	23.98±3.27	23.39±3.15	0.395
Sex (Male/Female)	25/38	16/17	-
Smoking (Never/Yes)	39/24	22/11	-
Drinking (Never/Yes)	44/19	20/13	-
Glucose (mmol/L)	5.20±0.35	5.84±1.28	0.008
Sleep score	8.11±0.99	11.61±1.58	-
Total cholesterol (mmol/L)	5.21±0.40	5.21±0.26	0.951
Triglyceride (mmol/L)	2.23±0.89	2.12±0.70	0.541
ALT (U/L)	32.49±31.65	31.75±23.97	0.907
AST (U/L)	31.76±26.18	32.63±20.71	0.868
Uric acid (μmol/L)	341.17±87.79	343.52±90.45	0.903
Folic acid (nmol/L)	17.98±4.33	16.59±6.89	0.299
Telomere length (AFU)	7.72±0.53	7.11±1.20	0.008

Supplementary Table 7. Demographic characteristics of study population divided into four groups according to the both sleep score and blood folic acid concentration. Clinical parameters are shown as Mean ± SD.

Characteristics	Low level of folic acid		High level of folic acid		*P* value
	Good sleep (N=26)	Poor sleep (N=21)	Good sleep (N=37)	Poor sleep (N=12)	
Age (years)	58.31±10.53	58.52±9.22	55.76±9.44	59.17±14.07	0.633
BMI (kg/m^2)	24.74±3.27	24.03±2.82	23.45±3.22	22.28±3.51	0.144
Sex (Male/Female)	13/13	11/10	12/25	5/7	-
Smoking (Never/Yes)	57.7%/42.3%	52.4%/47.6%	64.9%/35.1%	58.3%/41.7%	-
Drinking (Never/Yes)	57.7%/42.3%	57.1%/42.9%	75.7%/24.3%	66.7%/33.3%	-
Glucose (mmol/L)	5.44±0.31	6.37±1.32	5.03±0.27	4.92±0.39	< 0.001
Sleep score	8.04±1.08	11.62±1.72	8.17±0.93	11.58±1.39	< 0.001
Total cholesterol (mmol/L)	5.21±0.27	5.25±0.26	5.21±0.48	5.14±0.25	0.892
Triglyceride (mmol/L)	2.15±0.51	2.28±0.79	2.29±1.08	1.83±0.42	0.382
ALT (U/L)	31.85±22.69	35.43±28.15	32.95±36.98	25.33±12.72	0.817
AST (U/L)	31.15±21.94	33.19±20.95	32.19±29.09	31.67±21.19	0.994
Uric acid (µmol/L)	353.31±92.49	342.10±83.47	332.65±84.58	346.00±105.47	0.838
Folic acid (nmol/L)	14.03±3.15	12.32±3.98	20.75±2.50	24.08±3.62	< 0.001
Telomere length (AFU)	7.38±0.44	6.50±1.03	7.96±0.45	8.17±0.59	< 0.001

Supplementary Table 8. Linear regression analysis of the correlation between the leukocyte telomere length (AFU) and general characteristics.

Characteristics	Correlation coefficient	*P* value
Age	-0.020	0.001**
BMI	-0.022	0.760
Smoking	-0.042	0.559
Drinking	-0.026	0.713
Total cholesterol	-0.010	0.921
Triglyceride	-0.170	0.024*
Uric acid	-0.118	0.099
AST	-0.121	0.086
ALT	-0.080	0.254
Folic acid	0.095	< 0.001***
